# A Case of Long-Term Undiagnosed Cyclodialysis Cleft Following Tanito Microhook Trabeculotomy

**DOI:** 10.7759/cureus.77516

**Published:** 2025-01-15

**Authors:** Hinako Ohtani, Akiko Harano, Sho Ichioka, Ayaka Shimada, Mizuki Iida, Kana Murakami, Chisako Ida, Masaki Tanito

**Affiliations:** 1 Department of Ophthalmology, Shimane University Faculty of Medicine, Izumo, JPN

**Keywords:** anterior segment optical coherence tomography (as-oct), cyclodialysis cleft, direct internal cyclopexy, goniotomy, minimally invasive glaucoma surgery (migs), persistent hypotony, prolonged hypotony, tanito microhook trabeculotomy (tmh)

## Abstract

Persistent hypotony following Tanito microhook trabeculotomy (TMH) is rare but may occur due to the development of cyclodialysis clefts. We report a case of a Japanese man in his 40s who developed persistent hypotony and hypotony maculopathy after TMH in the left eye. Fourteen months after the surgery, the patient was referred to our institution due to prolonged hypotony that remained undiagnosed and untreated despite evaluations with gonioscopy and anterior segment optical coherence tomography (AS-OCT) at the referring clinic. At our institution, default AS-OCT scans and gonioscopy also failed to identify the cleft due to anterior iris rotation caused by ciliary body detachment. However, manual AS-OCT scans successfully revealed angle detachment and irido-trabecular contact at 203°. The patient underwent combined cataract surgery and direct internal cyclopexy to repair the cyclodialysis cleft. While the surgery successfully resolved hypotony, it resulted in elevated intraocular pressure, necessitating Ahmed glaucoma valve implantation. This case emphasizes the importance of detailed AS-OCT scanning in detecting subtle cyclodialysis clefts and highlights direct internal cyclopexy as an effective treatment option for prolonged hypotony after TMH.

## Introduction

Tanito microhook trabeculotomy (TMH) is a type of minimally invasive glaucoma surgery (MIGS) categorized as goniotomy, similar to Kahook Dual Blade (KDB; New World Medical, Inc., Rancho Cucamonga, CA) and Trabectome (NeoMedix Corp., Tustin, CA) [1]. Although transient hypotony below 5 mmHg has been reported in 17 of 679 eyes (1.0%) [2] and in 24 of 1,878 eyes (1.3%) [3] one day after a Trabectome procedure, prolonged hypotony after goniotomy is relatively rare, with an incidence of 0.7% reported following TMH [4].

We previously reported four cases of prolonged hypotony following TMH [5] and two cases following KDB [6]. In these cases, cyclodialysis cleft was observed at the goniotomy site via gonioscopy, and communication between the anterior chamber and suprachoroidal space was identified using ultrasound biomicroscopy (UBM) or anterior segment optical coherence tomography (AS-OCT). Consequently, determining the cause of hypotony in such cases is generally straightforward.

Herein, we present a case of prolonged hypotony following TMH performed at another institution, where cyclodialysis cleft remained undetected for an extended period before referral to our facility. We discuss the nuances of detecting cyclodialysis cleft using AS-OCT and the treatment approach utilizing direct internal cyclopexy [6,7].

## Case presentation

A Japanese man in his 40s underwent TMH in the right eye six years ago and in the left eye 14 months ago at an ophthalmology clinic. The TMH was performed nasally using the straight-type Tanito microhook (M2215-S, Inami Co. Ltd., Tokyo, Japan) without simultaneous cataract surgery. The preoperative intraocular pressure (IOP) of the left eye was 43 mmHg, which decreased immediately after surgery and remained at approximately 5 mmHg thereafter. The preoperative visual acuity (VA) of the left eye was 1.2, which remained stable at 1.2 for four months postoperatively but later declined to 0.3 due to hypotony maculopathy. Cyclodialysis cleft was not observed by gonioscopy, and AS-OCT revealed ciliary body detachment without evidence of communication between the anterior chamber and the suprachoroidal space.

During the follow-up, treatments such as continuous topical betamethasone phosphate, anterior chamber injection of viscoelastic material, air injection, and laser treatment to the peripheral nasal iris were performed; however, IOP did not improve. The patient was referred to our institution for the evaluation and treatment of prolonged hypotony.

**Figure 1:**
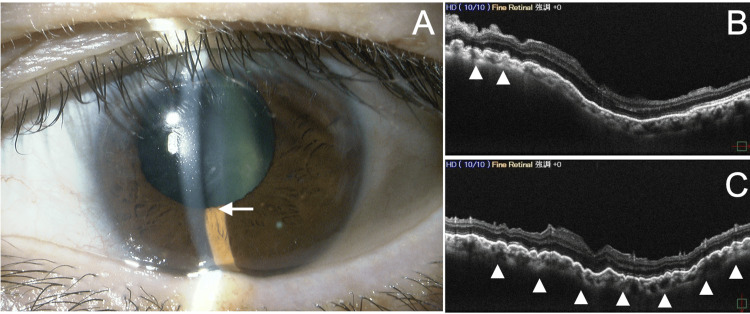
Slit lamp (A) and fundus optical coherence tomography (OCT) findings (B, C) 14 months after Tanito microhook trabeculotomy (TMH) (left eye). (A) Mild shallow anterior chamber and pupillary deformation are observed (arrow). (B, C) Vertical (B) and horizontal (C) scans of the macula by fundus OCT (RS-3000 Advance 2, Nidek, Gamagori, Japan) reveal the formation of macular retinal-choroidal folds (arrowheads).

**Figure 2:**
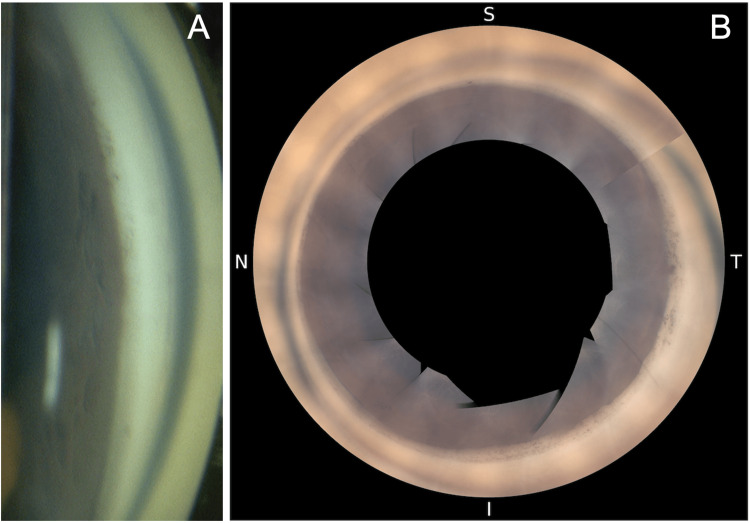
Gonioscopic findings (A) and 360° gonio-photography (B) of the nasal angle 14 months after Tanito microhook trabeculotomy (TMH) (left eye). (A) Gonioscopy shows no evidence of cyclodialysis cleft in the nasal angle, where goniotomy was performed. (B) 360° gonio-photography with GS-1 (Nidek, Gamagori, Japan) also reveals no cyclodialysis cleft. I, inferior; N, nasal; S, superior; T, temporal.

**Figure 3:**
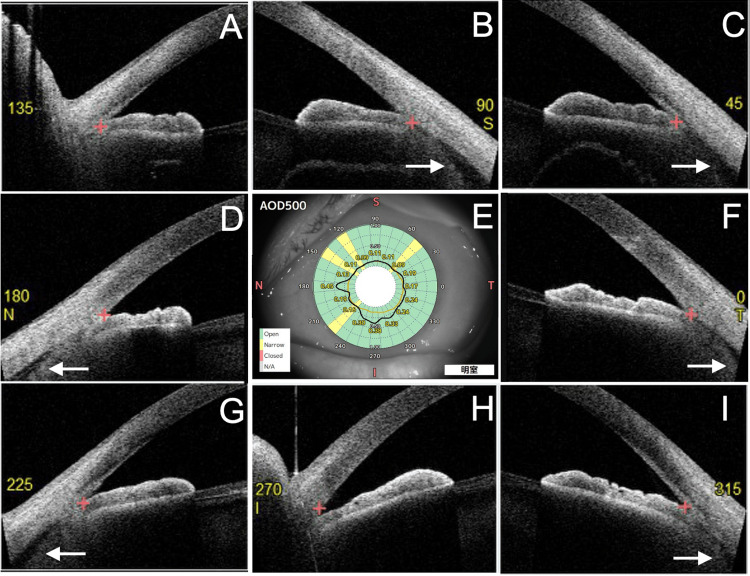
Findings of angle observation by anterior segment optical coherence tomography (OCT). (A-I) Using the default angle observation mode (STAR360) of Casia 2 (Tomey Corporation, Nagoya, Japan), a 45° interval scan detects ciliary body detachment (arrows) but no cyclodialysis cleft.

**Figure 4:**
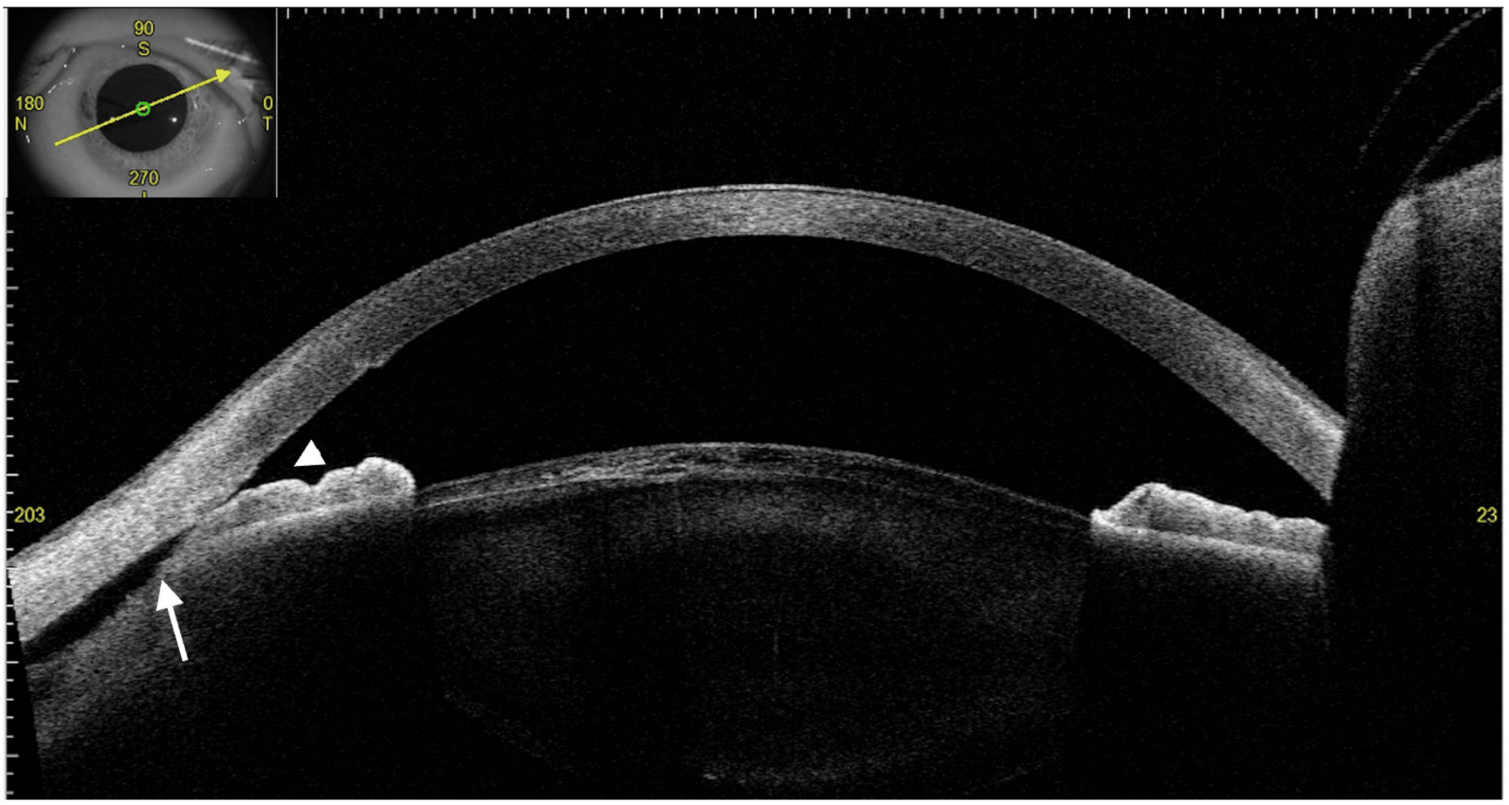
Anterior segment optical coherence tomography (AS-OCT) findings using a manually adjusted scanning axis. AS-OCT in the 23°-203° direction reveals an angle detachment cleft in the inferonasal quadrant (203°) (arrow) and irido-trabecular contact due to anterior iris rotation (arrowhead). The inset in the upper left shows the scanning direction.

At the initial visit to our hospital, the VA was 0.04 (1.2 × S-5.00D = C-0.50D Ax100°) in the right eye and 0.03 (0.4 × S-3.00D) in the left eye. IOP measured by a Goldmann tonometer was 15 mmHg in the right eye and 7 mmHg in the left eye. The right eye was on four glaucoma medications, and the left eye was treated with 0.1% betamethasone phosphate four times daily. Slit-lamp examination revealed a slightly shallower anterior chamber and mild posterior sub-capsular opacity in the left eye compared to the right (Figure 1A). Fundus OCT (RS-3000 Advance 2, Nidek, Gamagori, Japan) showed retinal-choroidal folds in the macula of the left eye (Figures 1B-1C). Gonioscopy (Figure 2A) and 360° gonio-photography (GS-1, Nidek, Gamagori, Japan) (Figure 2B) revealed no apparent cyclodialysis cleft. Using the angle observation mode (STAR360) of AS-OCT (Casia 2, Tomey Corporation, Nagoya, Japan), 45° interval scans showed ciliary body detachment throughout the angle but no communication between the anterior chamber and the suprachoroidal space (Figures 3A-3I). In manual AS-OCT scans, angle detachment at 203° and irido-trabecular contact due to anterior iris rotation were observed (Figure 4). The left eye was diagnosed with prolonged hypotony secondary to cyclodialysis cleft induced by TMH, cataract, and visual impairment from hypotony maculopathy.

**Figure 5:**
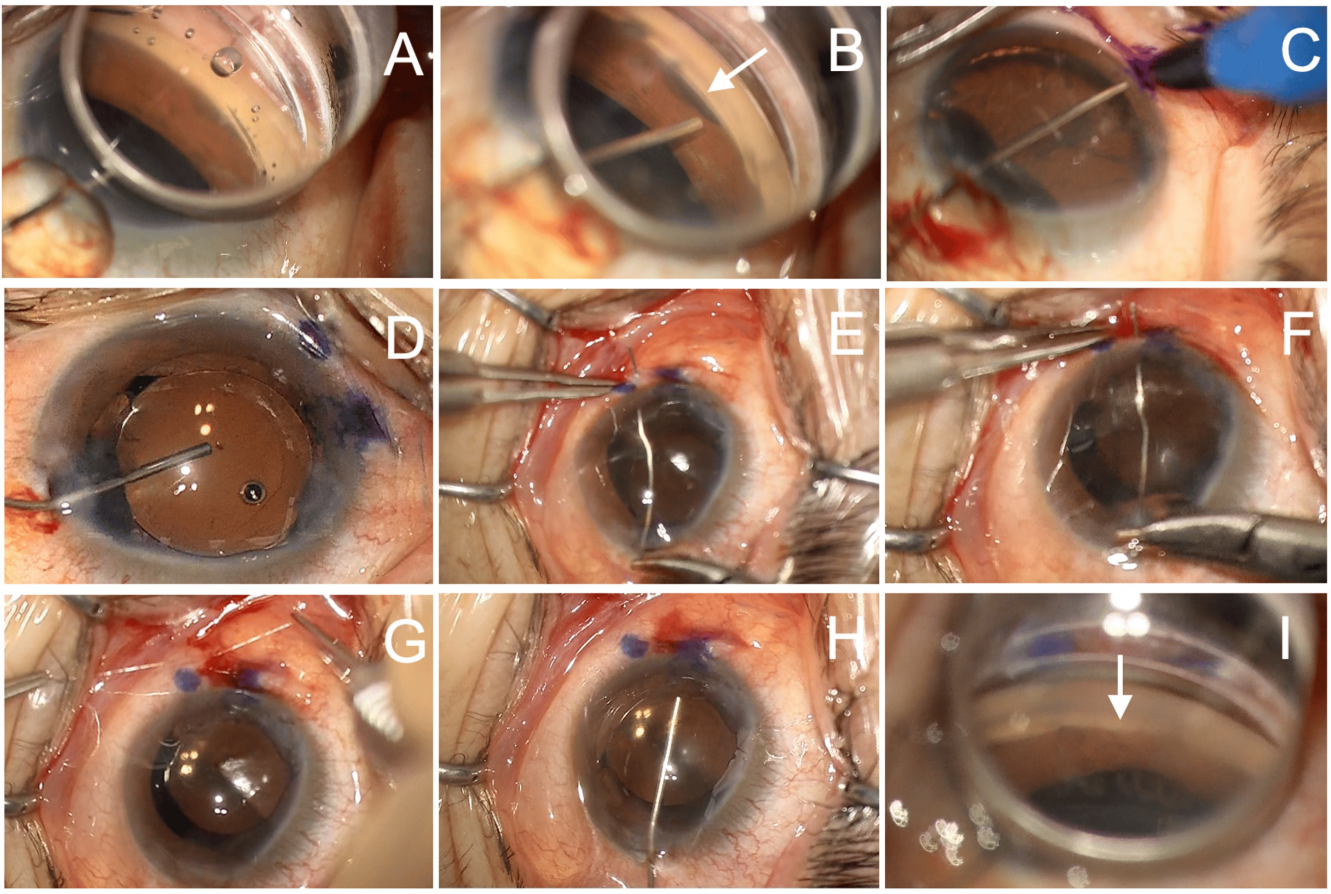
Repair of cyclodialysis cleft using direct internal cyclopexy combined with micro-incision cataract surgery. (A) Gonioscopic observation of the nasal angle. (B) Cyclodialysis cleft is observed (arrow) after injecting viscoelastic material into the anterior chamber. (C) The extent of the cyclodialysis cleft is marked using a marker pen. (D) Micro-incision cataract surgery is performed through a nasal corneal incision, followed by intraocular lens implantation. The ciliary sulcus is expanded with viscoelastic material. (E, F) Two needles of a 10-0 polypropylene suture (PC-9, Alcon Laboratories, Fort Worth, TX) are prepared. The suture is passed through the sclera and ciliary sulcus at locations covering the cyclodialysis cleft. (G) The suture is tied and rotated to bury the knot beneath the sclera without conjunctival incision. (H) Additional viscoelastic material is injected into the anterior chamber. (I) Successful repair of the cyclodialysis cleft is confirmed (arrow).

The patient traveled by plane from a distant area to visit our institution. Therefore, surgery was performed on the day of his visit following hospitalization. Cataract surgery combined with direct internal cyclopexy [6,7] was performed (Figures 5A-5I, Video 1). The axial length was 27.92 mm in the right eye and 25.70 mm in the left eye. To account for the potential postoperative myopic shift due to axial length recovery, a lower myopic target (-4D) was chosen, and a +23.0D intraocular lens (IOL; XY-1, Hoya Corporation, Tokyo, Japan) was implanted.

**Video 1:** Direct internal cyclopexy.

Standard cataract surgery was performed under topical proparacaine anesthesia with 2% lidocaine Tenon’s block after preoperative mydriasis. Using a Swan-Jacob gonio lens (Ocular Instruments, Bellevue, WA), the nasal angle was observed, and viscoelastic material (Provisc, Alcon Laboratories, Fort Worth, TX) was injected into the anterior chamber to reposition the anteriorly rotated iris, exposing the cyclodialysis cleft (Figures 5A-5B). The extent of the cleft was marked at the corneal limbus (Figure 5C). A 2.2-mm corneal incision was created nasally, and cataract surgery was completed. After IOL implantation into the capsular bag, the ciliary sulcus was expanded with viscoelastic material (Figure 5D). A 10-0 polypropylene suture (PC-9, Alcon Laboratories, Fort Worth, TX) was tied, and the needle was used to pass through the ciliary sulcus and sclera at two points covering the cyclodialysis cleft (Figures 5E-5F). Without conjunctival incision, the suture was tied and buried beneath the sclera by rotating the knot (Figure 5G). The viscoelastic material was re-injected to confirm cleft closure (Figures 5H-5I). Viscoelastic material was aspirated, and corneal incisions were sealed with hydration. A subconjunctival injection of 1 mg dexamethasone and topical levofloxacin ointment was applied to complete the procedure. Postoperative care included topical 1.5% levofloxacin and 0.1% betamethasone four times daily.

**Figure 6:**
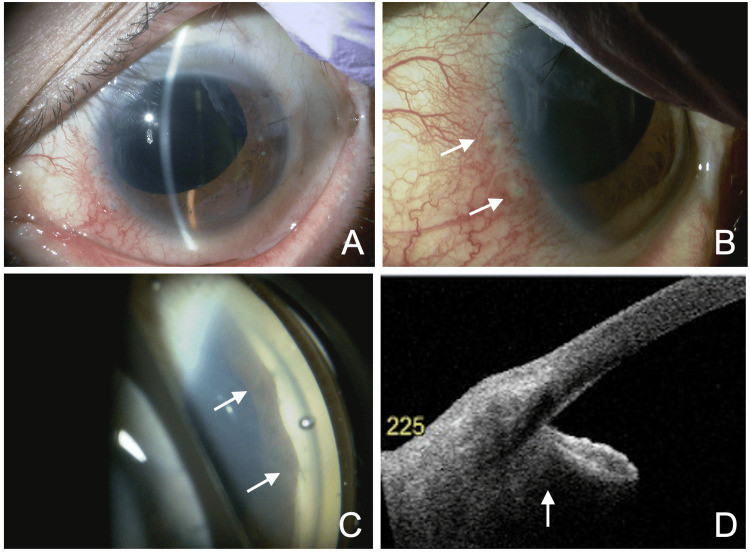
Findings 14 days after direct internal cyclopexy. (A) Slit lamp observation shows a deeper anterior chamber. Intraocular pressure (IOP) is 35 mmHg (under four glaucoma medications). (B) The suture tied on the conjunctiva has naturally buried itself into the subconjunctival space. (C) Gonioscopic observation shows the suture insertion site (arrows). (D) Anterior segment OCT shows that the iris root is pulled into the anterior chamber (arrow), confirming the repair of the cyclodialysis cleft.

**Figure 7:**
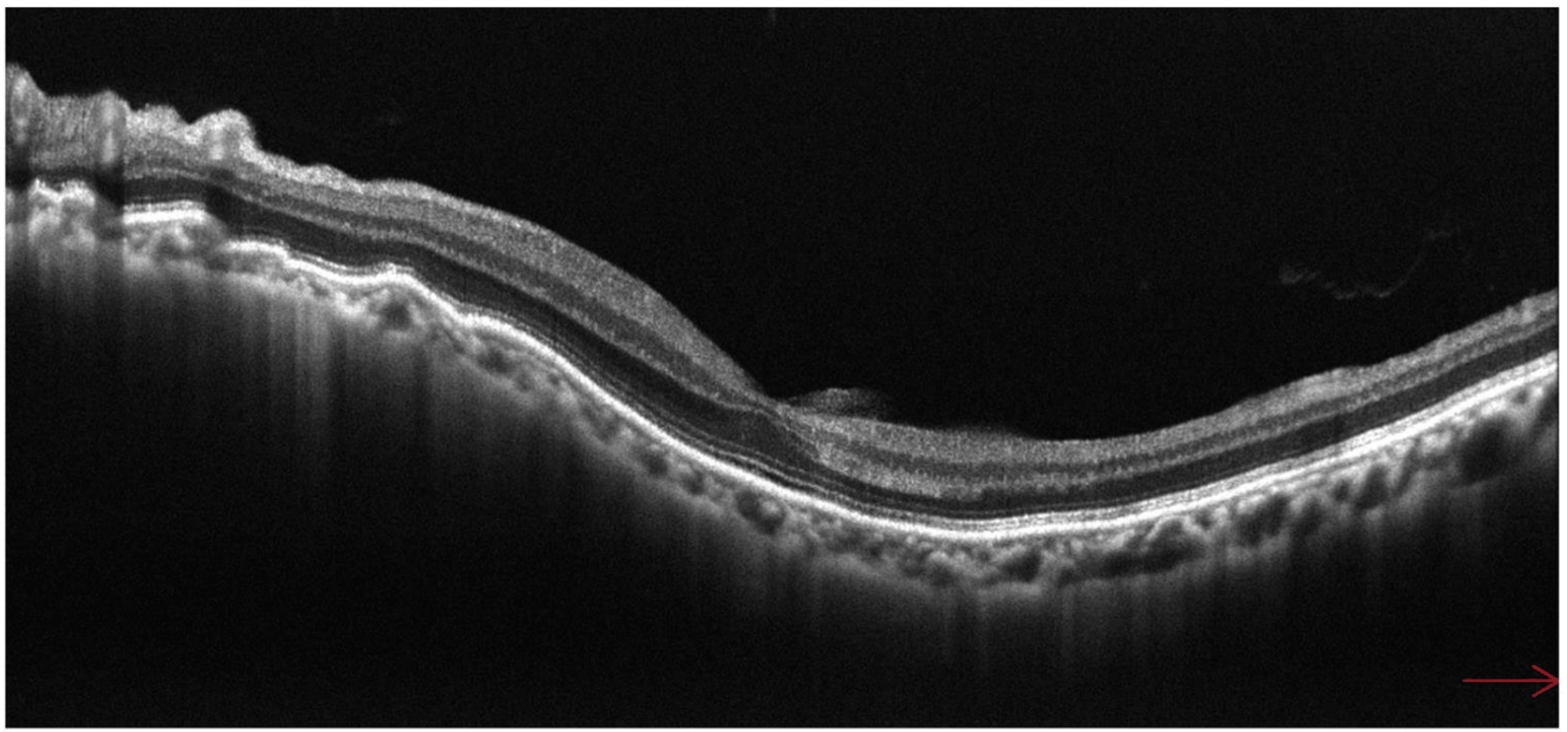
Fundus optical coherence tomography (OCT) findings 14 days after direct internal cyclopexy. Retinal-choroidal folds have significantly improved.

On the first postoperative day, IOP was 6 mmHg, and the anterior chamber depth improved. By the third postoperative day, VA was 0.01 (0.7 × -5.5D = C-3.0D Ax110°), and IOP was 44 mmHg with four IOP-lowering medications. At 14 days, IOP was 35 mmHg, the anterior chamber depth remained improved without significant inflammation (Figure 6A), and the suture had naturally buried itself under the conjunctiva (Figure 6B). Gonioscopy revealed the suture sites (Figure 6C), and AS-OCT confirmed the successful repair of the cyclodialysis cleft (Figure 6D). Retinal-choroidal folds significantly resolved (Figure 7).

Due to elevated IOP, Ahmed glaucoma valve (AGV, Model FP-7, New World Medical, Inc., Rancho Cucamonga, CA) insertion was performed on the same day. The AGV plate was fixed in the superotemporal sclera 8.5 mm from the limbus, and the tube tip was inserted into the ciliary sulcus [8]. Postoperatively, IOP was 11 mmHg with a deep anterior chamber.

**Figure 8:**
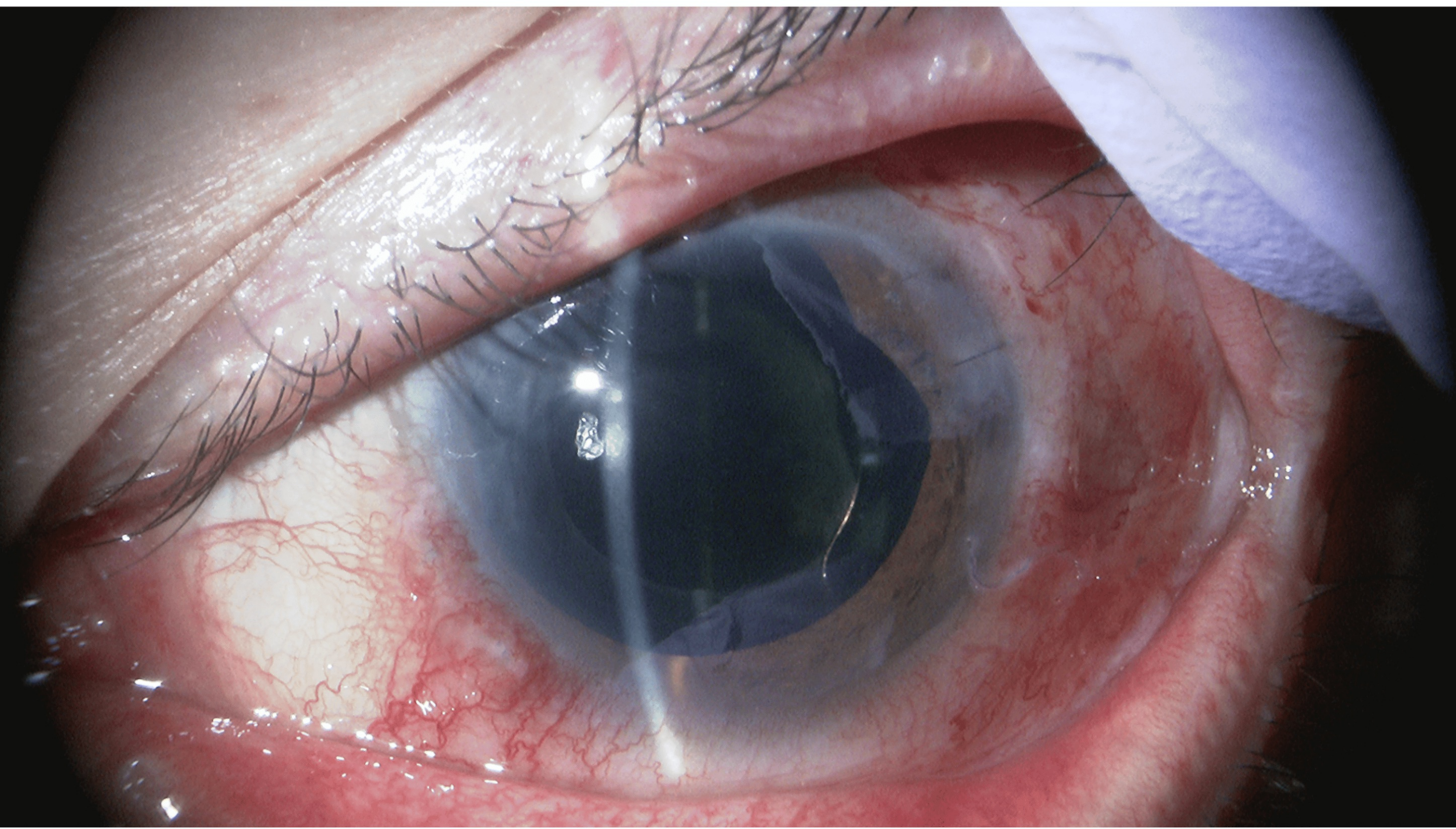
Slit lamp findings 14 days after ciliary sulcus insertion of Ahmed glaucoma valve (28 days after direct internal cyclopexy). The anterior chamber remains deep. Intraocular pressure (IOP) is 15 mmHg without glaucoma medications.

At 14 days post-AGV insertion (28 days post-cyclopexy), the anterior chamber remained deep without inflammation (Figure 8), and hypotony maculopathy did not recur. The VA of the left eye was 0.02 (0.5 × S-8.0D = C-0.75D Ax120°), and IOP was 15 mmHg. Two months later, at the referring clinic, the left eye VA had improved to 1.0, and IOP was 20 mmHg, reducing to 15 mmHg with massage.

## Discussion

This case highlights a challenging scenario where prolonged hypotony following TMH was not diagnosed or treated effectively due to the inability to detect a cyclodialysis cleft with gonioscopy or AS-OCT at the referring clinic. Even at our institution, initial evaluations with gonioscopy and default-mode AS-OCT failed to reveal the cleft. The anterior rotation of the iris caused by ciliary body detachment obscured the cleft during gonioscopy. Although attempts were made to compress the limbus with a cotton swab during gonioscopy, the cyclodialysis cleft remained undetected. However, manual AS-OCT scans successfully identified the site of angle damage. It is important to note that goniotomy-induced cyclodialysis clefts may be missed in default 45° interval scans if the affected site is not included. UBM allows the examiner to dynamically adjust the scanning position and provides greater tissue penetration, which might have been useful for understanding the pathology in this case.

The formation of cyclodialysis clefts during goniotomy procedures, including TMH, is thought to be the primary contributor to prolonged hypotony [5,6,9]. Previous studies suggest that factors such as younger age and myopia may increase the risk [5,6,9]. In this case, these risk factors were present. The pectinate ligament (also referred to as the ciliary process or mesodermic remnant) overlays the trabecular meshwork (TM). It has been proposed that pectinate ligament degeneration, a characteristic of juvenile open-angle glaucoma (JOAG), can occur with aging [10]. Additionally, glaucomatous TM is known to stiffen and lose elasticity over time [11,12]. Traction exerted on the pectinate ligament or TM by a blunt instrument, such as a microhook, could explain the development of clefts in younger glaucoma patients. Furthermore, higher grades of ciliochoroidal detachment have been linked to greater axial elongation after Trabectome procedures [13]. Although the precise mechanisms are not yet fully understood, structural changes associated with myopia may play a role in the development of cyclodialysis clefts. Myopia is also a well-documented risk factor for hypotony maculopathy after trabeculectomy [14], which likely contributed to the visual deterioration seen in this case.

Following the repair of the cyclodialysis cleft, a significant rise in IOP occurred, likely due to reduced perfusion of Schlemm’s canal and impaired conventional aqueous outflow [15]. This highlights the need to inform patients about the potential for IOP spikes after surgery and the possibility of requiring additional procedures to manage elevated IOP. As anticipated, our patient experienced substantial IOP elevation immediately after cyclopexy, necessitating AGV implantation. While trabeculectomy could have been an alternative, we chose AGV surgery for several reasons: avoiding the risk of recurrent hypotony maculopathy, reducing the need for frequent follow-ups for this long-distance patient, and addressing the challenges posed by associated uveitis, which could compromise bleb function in trabeculectomy cases.

Cyclodialysis clefts that arise postoperatively or after trauma may resolve spontaneously in some cases [5,16,17]. However, surgical intervention becomes necessary when conservative treatments fail [18,19]. Various techniques, including ab externo and ab interno approaches for ciliary body suturing, have been described [18,19]. Direct internal cyclopexy, utilizing an ab interno approach, minimizes ocular tissue manipulation by avoiding conjunctival or scleral incisions and vitreous handling. Nonetheless, it is important to note that this technique can only be performed in eyes with IOL implantation, as phakic eyes are unsuitable for this procedure.

## Conclusions

This case demonstrates the importance of suspecting cyclodialysis cleft in cases of prolonged hypotony after goniotomy, even when findings on gonioscopy and AS-OCT fail to show clear evidence of anterior chamber-to-suprachoroidal communication. Additionally, direct internal cyclopexy proved to be an effective technique for repairing goniotomy-induced cyclodialysis clefts, emphasizing its utility in managing such challenging cases.
